# Supplementary material for: Mutations in apicoplast rRNA genes are associated with clindamycin resistance and impair the ability of malaria parasites to infect mosquitoes
Source: Antimicrob Agents Chemother. 2026 Jul 15;70(8):e00507-26. doi: 10.1128/aac.00507-26 (PMC13436215; doi:10.1128/aac.00507-26)
Supplement: Supplementary file 1 [file aac.00507-26-s0001.docx]

**Figure S1**

Nucleotide sequence alignment of portion of the large ribosomal subunit RNA gene in *Plasmodium falciparum* (CP131995.1)*, Plasmodium berghei* (AB649421.1)*, Toxoplasma gondii* (NC_001799.1)*, Chromera velia* (KM062125.1) and *Escherichia coli* (CP032679.1)*.* Purple colouration signifies nucleotide conservation between species. Red outline indicates identified mutated residues (*E.coli* G2447, G2576, G2581) in selected *P. falciparum* clindamycin resistant lines. Multiple sequence alignment was generated using EMBL-EBI Clustal Omega and edited using Jalview.

**Figure S2**

Example sequence contigs of apicoplast large ribosomal subunit rRNA (LSU) inverted repeat A and B in *Pf*NF54_Cl^R^_S and *Pf*NF54_Cl^R^_P lines. Arrows indicate primer pairs used to amplify inverted repeat A (blue) and inverted repeat B (green) for amplicon sequencing.

|  | **Antimalarial** | ***Pf*NF54_WT** | | ***Pf*NF54_Cl^R^_S** | | **Known/putative target and mechanism of action** |
| --- | --- | --- | --- | --- | --- | --- |
|  |  | **n** | **IC50 (mean ± SD) (μM)** | **n** | **IC50 (mean ± SD) (μM)** |  |
| **144 hr assay** | **Clindamycin** | 5 | 0.008 ± 0.0015 | 5 | 0.166 ± 0.0429 | Apicoplast 23S rRNA, peptidyl transferase activity (1, 2) |
|  | **Chloramphenicol** | 2 | 5.716 ± 1.7140 | 2 | 2.865 ± 0.6416 | Apicoplast 23S rRNA, peptidyl transferase activity (3, 4) |
|  | **Azithromycin** | 2 | 0.038 ± 0.0014 | 2 | 0.018 ± 0.0018 | Apicoplast 23S rRNA, peptide elongation (5, 6) |
|  | **Doxycycline** | 2 | 0.613 ± 0.0815 | 2 | 0.310 ± 0.0437 | Apicoplast 30S small ribosomal subunit, aminoacyl tRNA binding at A-site (7, 8) |
| **96 hr assay** | **Fosmidomycin** | 2 | 1.606 ± 0.1902 | 2 | 1.220 ± 0.1997 | Apicoplast, DOXP reductoisomerase, isoprenoid biosynthesis  (9, 10) |
|  | **Atovaquone** | 2 | 0.001 ± 0.0002 | 2 | 0.001 ± 0.0003 | Mitochondria, cytochrome *b*, electron transport chain (11) |
|  | **Pyrimethamine** | 2 | 0.037 ± 0.0121 | 2 | 0.033 ± 0.0013 | DHFR, folate synthesis (12) |

**Table S1**

Cross-resistance *in vitro* drug assays performed on *Pf*NF54_Cl^R^_S. Mean half-maximal inhibitory concentration (IC50 (μM)) and standard deviation (SD) recorded from ‘n’ biological replicates. Putative targets and mechanism/s of action of antimalarials are based on either *Plasmodium* or bacterial studies

| *Pf*NF54_Cl^R^_S/P apicoplast 23S rRNA forward inverted repeat | Forward | 5’-TAGTTTGACTGGGGCGGTCTCC-3’ |
| --- | --- | --- |
|  | Reverse | 5’-ACCACAATTCTTCATCTTAACCA-3’ |
| *Pf*NF54_Cl^R^_S/P apicoplast 23S rRNA reverse inverted repeat | Forward | 5’-TAGTTTGACTGGGGCGGTCTCC-3’ |
|  | Reverse | 5’-ACGTTCAAATTGTGCAAAATCAGA-3’ |
| *Pf*Dd2_Cl^R^_S/P apicoplast 23S rRNA | Forward | 5’-CTTAAGGAATTGAATCATCTTAG-3’ |
|  | Reverse | 5’-CAACTTATTAGGAATTATACAC-3’ |

**Table S2**

Primers for PCR amplification and sequencing.

**Supplementary References:**

1.	Dharia NV, Plouffe D, Bopp SER, González-Páez GE, Lucas C, Salas C, Soberon V, Bursulaya B, Kochel TJ, Bacon DJ, Winzeler EA. 2010. Genome scanning of Amazonian *Plasmodium falciparum* shows subtelomeric instability and clindamycin-resistant parasites. Genome Res 20:1534–1544.

2.	Guay D. 2007. Update on clindamycin in the management of bacterial, fungal and protozoal infections. Expert Opinion on Pharmacotherapy 8:2401–2444.

3.	Uddin T, McFadden GI, Goodman CD. 2017. Validation of Putative Apicoplast-Targeting Drugs Using a Chemical Supplementation Assay in Cultured Human Malaria Parasites. Antimicrob Agents Chemother 62:e01161-17.

4.	Wilson DN. 2009. The A–Z of bacterial translation inhibitors. Critical Reviews in Biochemistry and Molecular Biology 44:393–433.

5.	Dahl EL, Rosenthal PJ. 2007. Multiple antibiotics exert delayed effects against the *Plasmodium falciparum* apicoplast. Antimicrob Agents Chemother 51:3485–3490.

6.	Parnham MJ, Haber VE, Giamarellos-Bourboulis EJ, Perletti G, Verleden GM, Vos R. 2014. Azithromycin: Mechanisms of action and their relevance for clinical applications. Pharmacology & Therapeutics 143:225–245.

7.	Dahl EL, Shock JL, Shenai BR, Gut J, DeRisi JL, Rosenthal PJ. 2006. Tetracyclines specifically target the apicoplast of the malaria parasite *Plasmodium falciparum*. Antimicrob Agents Chemother 50:3124–3131.

8.	Roberts MC. 1996. Tetracycline resistance determinants: mechanisms of action, regulation of expression, genetic mobility, and distribution. FEMS Microbiol Rev 19:1–24.

9.	Jomaa H, Wiesner J, Sanderbrand S, Altincicek B, Weidemeyer C, Hintz M, Türbachova I, Eberl M, Zeidler J, Lichtenthaler HK, Soldati D, Beck E. 1999. Inhibitors of the Nonmevalonate Pathway of Isoprenoid Biosynthesis as Antimalarial Drugs. Science 285:1573–1576.

10.	Kuzuyama T, Shimizu T, Takahashi S, Seto H. 1998. Fosmidomycin, a specific inhibitor of 1-deoxy-d-xylulose 5-phosphate reductoisomerase in the nonmevalonate pathway for terpenoid biosynthesis. Tetrahedron Letters 39:7913–7916.

11.	Fry M, Pudney M. 1992. Site of action of the antimalarial hydroxynaphthoquinone, 2-[trans-4-(4’-chlorophenyl) cyclohexyl]-3- hydroxy-1,4-naphthoquinone (566C80). Biochemical Pharmacology 43:1545–1553.

12.	Yuthavong Y. 2002. Basis for antifolate action and resistance in malaria. Microbes and Infection 4:175–182.
